# Prevention and Management of Dermatologic Adverse Events in Patients Treated with Amivantamab Plus Lazertinib

**DOI:** 10.3390/curroncol33020096

**Published:** 2026-02-04

**Authors:** Carolyn Szwed, Leszek Blicharz, Magdalena Knetki-Wroblewska, Lidia Rudnicka, Joanna Czuwara

**Affiliations:** 1 Department of Dermatology, Medical University of Warsaw, 02-006 Warsaw, Poland; carolyn.szwed@wum.edu.pl (C.S.); leszek.blicharz@wum.edu.pl (L.B.); lidia.rudnicka@wum.edu.pl (L.R.); 2 Department of Lung Cancer and Thoracic Tumors, Maria Sklodowska-Curie National Research Institute of Oncology, 02-781 Warsaw, Poland; magdalena.knetki-wroblewska@nio.gov.pl

**Keywords:** amivantamab plus lazertinib, amivantamab, lazertinib, dermatologic adverse events, EGFR inhibitors, non-small cell lung cancer, side effects

## Abstract

Amivantamab plus lazertinib (amivantamab+lazertinib) is a novel combination therapy used to treat epidermal growth factor receptor (EGFR)-mutated non-small cell lung cancer (NSCLC). Although this regimen has demonstrated clinical efficacy in locally advanced or metastatic NSCLC, it is associated with a range of dermatologic adverse events that may develop rapidly and require prompt intervention. Early management of these side effects is critical for maintaining oncologic treatment, optimizing clinical outcomes, and improving patients’ quality of life. **Cases:** We present four patients with EGFR-mutated NSCLC who experienced dermatologic adverse events during amivantamab+lazertinib therapy. Two developed severe facial papulopustular eruptions, and two presented with necrotic folliculitis with sanguineous scalp erosions. Additional dermatologic manifestations included paronychia, pruritus, xerosis, mucositis, and trichomegaly. Notably, one patient experienced milder dermatologic side effects due to early initiation of a dermatologic prophylactic regimen. **Conclusions:** Dermatologic adverse events during amivantamab+lazertinib therapy are frequent and may be more severe than those typically observed with EGFR inhibitors. Early prophylactic measures and timely intervention are essential for managing these adverse events, supporting treatment adherence, and maximizing the therapeutic efficacy of this combined oncologic regimen.

## Simple Summary

Amivantamab plus lazertinib (amivantamab+lazertinib) is an effective, novel treatment combination for epidermal growth factor receptor (EGFR)-mutated non-small cell lung cancer (NSCLC). Despite its efficacy, it often causes rapid onset dermatologic side effects that require prompt care. This article presents four such cases. Two patients developed severe papulopustular facial rashes, and two presented with severe folliculitis with scalp erosions. Additional findings included paronychia, pruritus, xerosis, mucositis, and trichomegaly. Notably, one patient experienced less severe side effects due to early use of a dermatologic prophylactic regimen. These cases suggest that dermatologic side effects associated with amivantamab+lazertinib may be more severe than those typically seen with EGFR inhibitors. Early preventive strategies and timely management are critical for supporting treatment adherence and optimizing clinical outcomes.

## 1. Introduction

Amivantamab plus lazertinib (amivantamab+lazertinib) is a novel combination therapy consisting of amivantamab, a fully human bispecific monoclonal antibody targeting epidermal growth factor receptor (EGFR) and mesenchymal–epithelial transition (MET), and lazertinib, a third-generation EGFR tyrosine kinase inhibitor (TKI) [1]. The combination was approved by the U.S. Food and Drug Administration (FDA) in 2024 for the first-line treatment of EGFR-mutated (exon 19 deletion or exon 21 L858R substitution) locally advanced or metastatic non-small cell lung cancer (NSCLC) [2]. Despite its clinical efficacy, this regimen is associated with a range of dermatologic adverse events [3].

Here, we present four patients with EGFR-mutated NSCLC who received amivantamab+lazertinib therapy. Two developed severe facial papulopustular eruptions, and two presented with necrotic folliculitis with sanguineous scalp erosions. Three patients exhibited additional milder dermatologic adverse events, such as paronychia, pruritus, xerosis, mucositis, and trichomegaly. Notably, one patient experienced less severe dermatologic side effects due to early initiation of a dermatologic prophylactic regimen.

## 2. Cases

### 2.1. Case I

**Figure 1:**
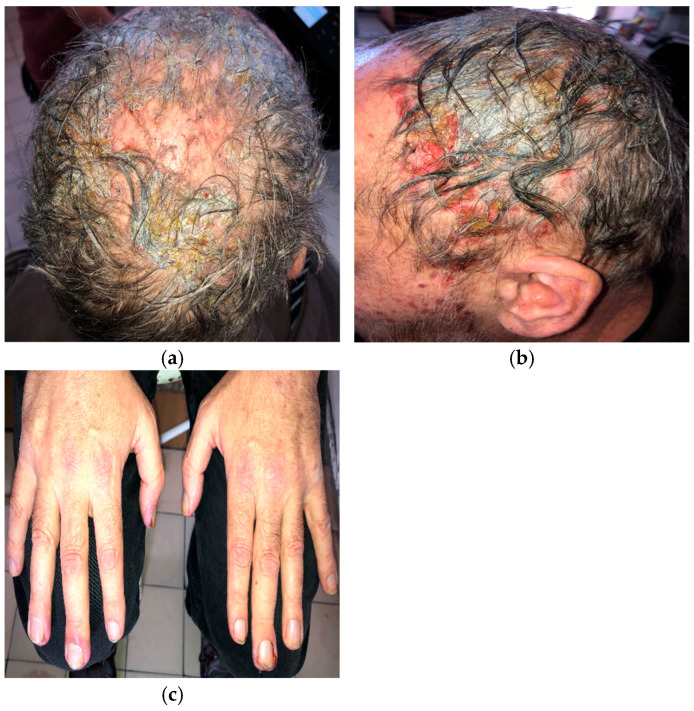
Necrotic folliculitis with sanguineous scalp erosions (**a**,**b**); paronychia associated with amivantamab+lazertinib therapy (**c**).

A 56-year-old man with EGFR-mutated (exon 19 deletion) metastatic NSCLC presented with severe (grade 3; NCI CTCAE v5.0) necrotic folliculitis (Figure 1a,b), paronychia (Figure 1c), pruritus, and xerosis, which developed two weeks after starting amivantamab+lazertinib therapy. His prior treatments included osimertinib and platinum-based chemotherapy, both of which failed to control disease progression. The patient also developed oral candidiasis and painful erosions on the buccal mucosa and palate.

Management of folliculitis included oral cefuroxime 500 mg twice daily combined with prednisone 20 mg daily, tapered to 5 mg over 10 days, followed by oral doxycycline 100 mg twice daily. Paronychia was treated topically with a moderate-potency corticosteroid combined with gentamicin. Silver dressings were applied interchangeably with iodine soaks. The patient was also advised to use ceramide-based moisturizers daily after bathing. Nystatin suspension three times daily was prescribed for oral candidiasis.

Amivantamab+lazertinib treatment was ultimately discontinued due to the severity of dermatologic adverse events.

### 2.2. Case II

**Figure 2:**
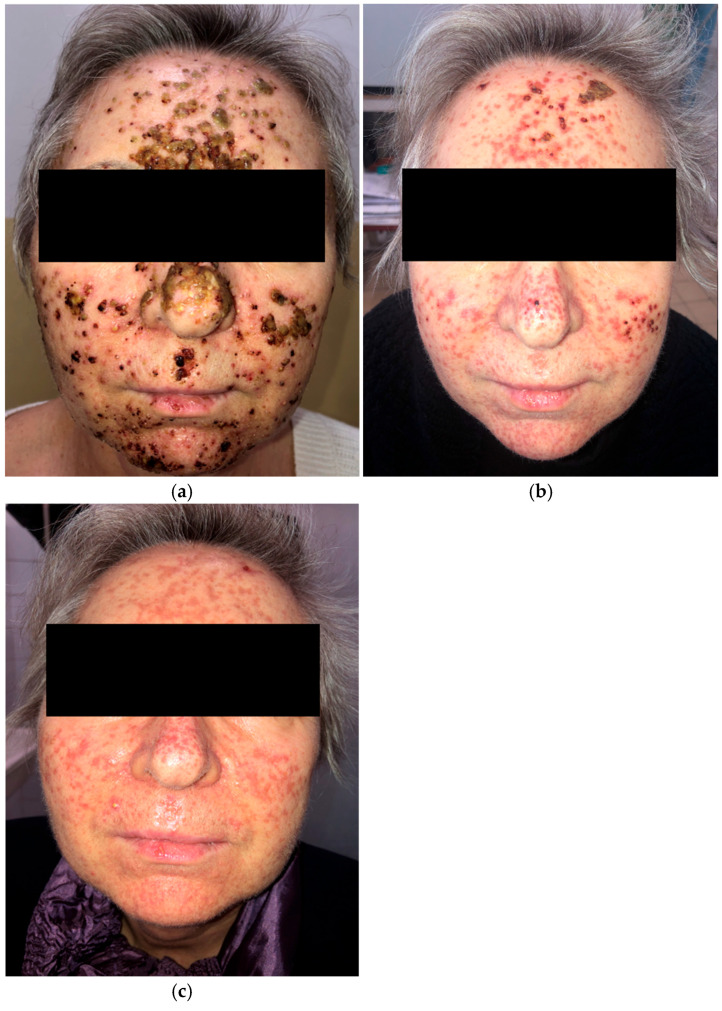
Papulopustular eruption of the face. (**a**) Initial presentation; (**b**) one month after treatment with oral cefuroxime, low-dose prednisone, and oral tetracycline; (**c**) two months after oral tetracycline and isotretinoin, introduced separately.

A 63-year-old woman receiving amivantamab+lazertinib for locally advanced EGFR-mutated NSCLC developed a rapidly progressive grade 3 (NCI CTCAE v5.0) papulopustular eruption on the face (Figure 2a) three weeks after initiating treatment. She also presented with trichomegaly of the eyelashes and painful vesicles and erosions on the lips. Initial management included oral cefuroxime 500 mg twice daily for 10 days, prednisone 10 mg daily, and topical clindamycin and ivermectin. She was subsequently treated with oral tetracycline 500 mg twice daily, and prednisone was discontinued.

The patient showed clinical improvement; however, residual facial scarring persisted after one month (Figure 2b). Two months later, she was started on low-dose isotretinoin (10 mg daily) following a one-week interval after tetracycline withdrawal (Figure 2c). Despite several weeks of tetracycline and isotretinoin therapy, the scarring remained.

Amivantamab+lazertinib treatment was briefly interrupted due to the development of grade 3 dermatologic adverse events but was later continued without dose modification following dermatologic management.

### 2.3. Case III

**Figure 3:**
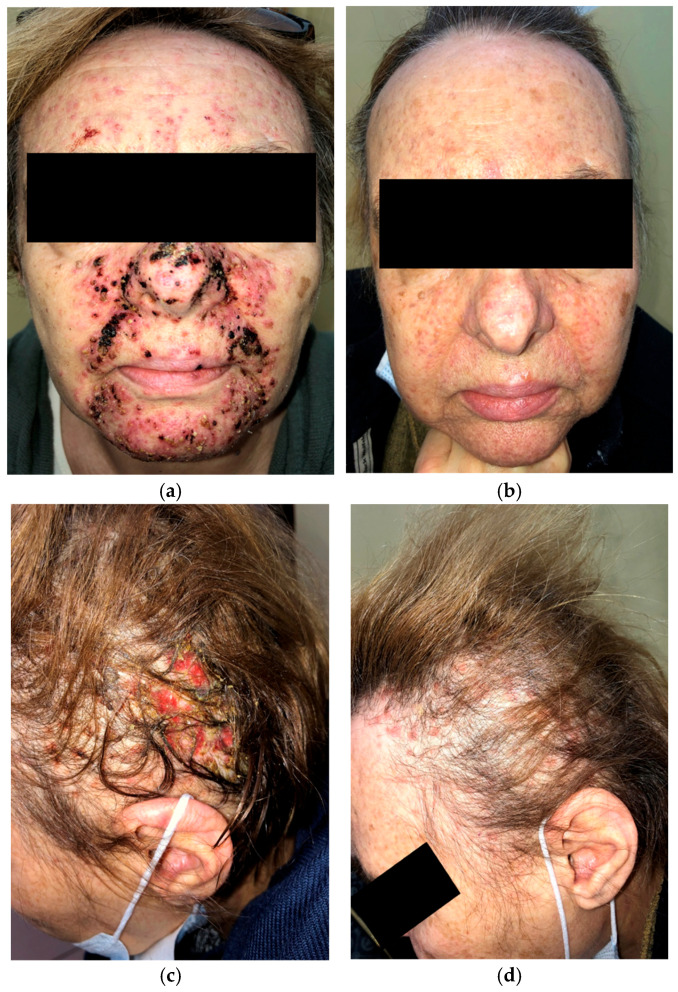
Initial presentation of papulopustular rash affecting the face (**a**); two months after oral cefuroxime and prednisone, followed by oral tetracycline with topical clindamycin and ivermectin (**b**); initial presentation of destructive folliculitis with scalp erosions (**c**); one month after oral cefuroxime, low-dose prednisone, and oral tetracycline interventional treatment (**d**).

A 63-year-old woman on amivantamab+lazertinib for metastatic EGFR-mutated NSCLC presented with a severe papulopustular facial rash (Figure 3a) and grade 3 (NCI CTCAE v5.0) folliculitis with scalp erosions (Figure 3c) two weeks after beginning treatment. She had previously received oral doxycycline without any clinical improvement. Management was escalated to oral cefuroxime 500 mg twice daily followed by oral tetracycline 500 mg twice daily. Systemic prednisone (10 mg daily) was administered during the first two weeks of therapy.

After two months of treatment with oral tetracycline combined with topical clindamycin and ivermectin, the papulopustular facial lesions showed marked improvement (Figure 3b). Destructive scalp folliculitis with erosions resolved after one month of combined oral cefuroxime and tetracycline therapy with low-dose prednisone (Figure 3d).

Treatment with amivantamab+lazertinib was not interrupted or discontinued.

### 2.4. Case IV

**Figure 4:**
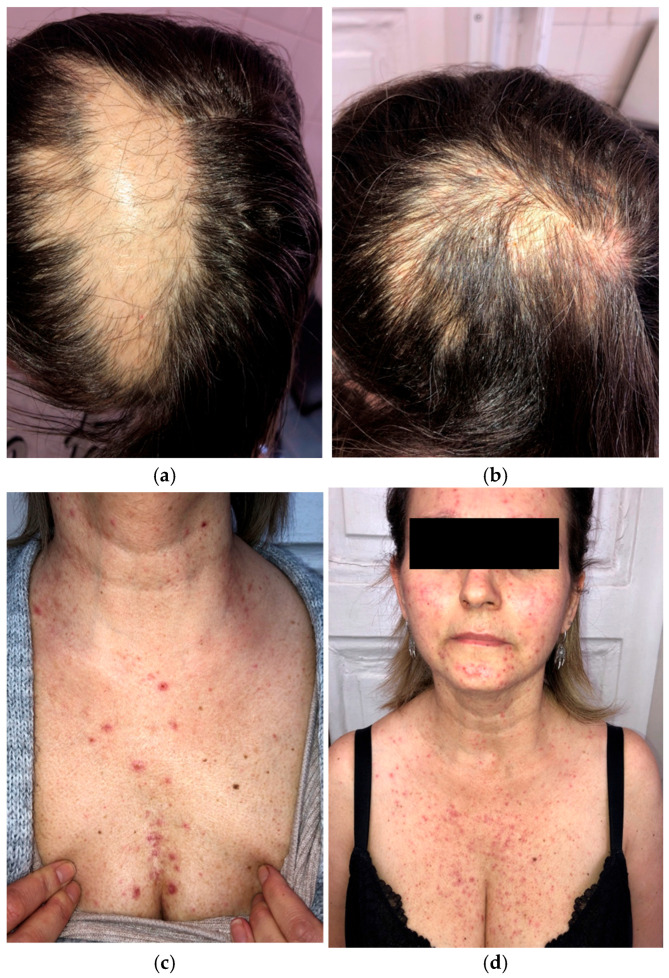
Initial presentation of scalp alopecia secondary to radiation therapy (**a**); two months after amivantamab+lazertinib treatment (**b**); grade 2 papulopustular rash while on dermatologic prophylactic treatment with oral tetracycline (**c**,**d**).

A 46-year-old woman preparing to begin treatment with amivantamab+lazertinib for metastatic EGFR-mutated NSCLC presented with scalp alopecia secondary to prior radiation therapy (Figure 4a,b). Her NSCLC had progressed despite treatment with afatinib, osimertinib, and platinum-based chemotherapy.

Given her history of dermatologic adverse events with previous therapies, a dermatologic prophylactic regimen, including oral tetracycline and topical clindamycin, was initiated before starting amivantamab+lazertinib. After four weeks of amivantamab+lazertinib treatment, she developed a grade 2 (NCI CTCAE v5.0) papulopustular eruption (Figure 4c,d) but did not experience the severe papulopustular eruptions observed in the other patients. Similarly, the patient did not develop severe necrotic folliculitis with scalp erosions. She experienced milder dermatologic side effects, such as eyelash trichomegaly and xerosis. The patient was able to continue the novel combination therapy without any grade ≥ 3 adverse events or dose modification.

## 3. Discussion

EGFR, a member of the receptor tyrosine kinase family, plays a crucial role in the maturation and differentiation of epidermal and epithelial cells, including adnexal structures such as hair follicles and sebaceous glands. EGFR is frequently overexpressed in many tumors, making its inhibitors effective and selective antineoplastic agents [4]. Disruption of EGFR signaling significantly affects keratinocyte homeostasis, leading to increased apoptosis and the recruitment of innate immune cells [4].

The most common and predictable dermatologic adverse event associated with EGFR inhibitors is an acneiform rash on the face, with a mean onset of approximately 1.5 weeks [5]. Other commonly affected anatomic sites include the chest, back, and scalp, which are rich in folliculosebaceous units. Facial lesions typically appear in central areas (forehead, nose, and chin) and resemble rosacea, often responding well to conventional rosacea treatments [6] (Table 1).

The term “acneiform rash” has been replaced with “papulopustular eruption,” which is considered more accurate [7]. The severity of papulopustular eruptions is graded from 1 to 5 according to NCI CTCAE v5.0, with grade 1 representing mild eruptions and grade 5 indicating death [6]. Other dermatologic side effects of anti-EGFR therapy include xerosis, pruritus, paronychia, periungual pyogenic granuloma, alopecia, and trichomegaly [7].

EGFR inhibition can be achieved through two main mechanisms. Monoclonal antibodies (e.g., cetuximab and panitumumab) bind to the extracellular domain of the receptor to prevent ligand-mediated activation. Small-molecule TKIs such as gefitinib, erlotinib, and afatinib act intracellularly to block the EGFR tyrosine kinase domain, thereby inhibiting downstream signaling cascades [4].

Inflammatory cutaneous adverse events, particularly involving the face, are frequent in patients receiving anti-EGFR therapy, with reported incidences ranging from 45% to 100% [4]. These reactions are well-recognized by both dermatologists and oncologists [7]. Several guidelines have been published on the management of dermatologic side effects associated with anti-EGFR therapy. Management usually involves a combination of interventions, including gentle skin care, moisturizers, photoprotection [8], and topical anti-inflammatory agents such as low-potency corticosteroids, ivermectin, and antibiotics (clindamycin, erythromycin, mupirocin, and metronidazole). In severe cases, prolonged systemic therapy with tetracyclines or isotretinoin may be indicated [9,10,11] (Table 1 and Table 2). Both topical and systemic corticosteroids should be used with caution [12].

Tetracyclines are generally well tolerated and exhibit multiple anti-inflammatory effects, including inhibition of IL-1 and TNF-α, suppression of metalloproteinase activity, reduction in monocyte and neutrophil chemotaxis, inhibition of angiogenesis, and downregulation of the cathelicidin antimicrobial peptide [14]. In dermatology, tetracyclines are widely used to treat chronic inflammatory conditions, such as acne vulgaris and rosacea, producing favorable clinical outcomes.

Histopathological studies of papulopustular eruptions induced by anti-EGFR therapies have demonstrated superficial, predominantly neutrophilic, suppurative folliculitis, often involving an enlarged infundibulum or ruptured epithelium of the upper portion of the affected hair follicle [15]. Routine histopathological evaluation of papulopustular eruptions affecting the pilosebaceous units is generally not required. The main concern is bacterial superinfection, which can exacerbate skin damage, as necrotic debris serves as a nidus for bacterial colonization [5]. This complication is time-sensitive, and delays in initiating appropriate antibiotic therapy may increase the risk of severe complications, particularly in immunocompromised or oncologic patients [12]. To prevent severe cutaneous adverse events, minimize delays in reactive treatment, avoid complications, and reduce the risk of oncologic therapy cessation, dermatologic prophylactic measures have been proposed [6] (Table 2).

Given that anti-EGFR therapies affect the skin in most patients and that dermatologic involvement may positively correlate with oncologic efficacy [7], it is important to consider the severity of adverse skin events associated with amivantamab+lazertinib therapy. We hypothesize that these adverse events arise from the more potent anti-EGFR activity of the combination, resulting in more pronounced inflammatory skin changes and tissue damage.

Amivantamab is a bispecific monoclonal antibody targeting EGFR and MET with unique mechanisms of action, including ligand blockade, receptor degradation, and recruitment of innate immune effector cells capable of tissue degradation [3]. Lazertinib is a third-generation EGFR TKI that selectively targets mutated EGFR, penetrates the central nervous system, and has a safety profile suitable for combination therapy [3]. The combination of amivantamab and lazertinib received FDA approval in 2024 for the treatment of locally advanced or metastatic EGFR-mutated NSCLC. This approval followed the MARIPOSA phase 3 clinical trial [3].

A total of 429 patients were enrolled in the MARIPOSA study and randomly assigned to receive amivantamab+lazertinib or osimertinib. Yang et al. presented the latest data on the efficacy and safety of the treatments. Following a median follow-up period of 37.8 months, the group of patients treated with amivantamab+lazertinib demonstrated a significant prolongation of overall survival (HR for death, 0.75; 95% CI, 0.61–0.92). The percentages of patients alive after three years of follow-up were 60% and 51% in the amivantamab+lazertinib and osimertinib groups, respectively. Combination therapy also significantly prolonged the time to symptomatic disease progression, with median durations of 49.3 versus 27.3 months (HR, 0.69; 95% CI, 0.57–0.83), and the median time to subsequent therapy was 33 versus 24 months (HR, 0.76; 95% CI, 0.64–0.90). Adverse events occurred more frequently with amivantamab+lazertinib, including grade 3 or higher events (80% versus 52% of participants) [16].

The complex mechanism of action of amivantamab leads to a wide spectrum of possible adverse reactions. The most common reactions are dermatologic complications, which are discussed in detail in this article. Other frequently observed reactions included infusion-related reactions (IRRs) (65%), hypoalbuminemia (51%), peripheral edema (38%), increased AST activity (40%), and diarrhea (32%). Among grade 3 or higher adverse events, pulmonary embolism was the most frequently reported. It should be emphasized that most adverse events occurred within the first four months of treatment [16]. Given this adverse event profile, prophylaxis against the most significant complications of amivantamab+lazertinib treatment is recommended. Prevention of dermatologic adverse events has been discussed in detail. Prophylaxis against IRRs is recommended through the use of corticosteroids for two days prior to and on the day of amivantamab administration, together with paracetamol and diphenhydramine. This approach significantly reduces the risk of IRRs (67.4% with standard prophylaxis versus 22.5% with an intensified regimen). Prophylaxis for thromboembolic complications with novel oral anticoagulants or low-molecular-weight heparin is also recommended for the first four months of treatment [17].

In our experience with 20 patients treated with amivantamab+lazertinib, nearly one-fifth required dermatologic consultation for severe skin rashes unresponsive to doxycycline. These observations suggest that dermatologic adverse events associated with this combination therapy are both time- and severity-dependent, and delays in initiating interventional treatment may reduce its effectiveness. Notably, one patient demonstrated that prophylaxis can mitigate the severity of these dermatologic adverse events, whereas three others experienced a reduced quality of life due to painful and disfiguring eruptions that were difficult to manage. Therefore, we propose that prophylactic management may reduce the severity, but not necessarily the incidence, of dermatologic adverse events associated with amivantamab+lazertinib.

Dermatologic adverse events related to amivantamab have been reported by Belzer et al. [18] and George et al. [19], although with differing terminology. Belzer et al. described patients with extensive necrotic scalp lesions as having an “atypical acneiform eruption,” which does not resemble classic acneiform folliculitis or folliculitis decalvans previously described with afatinib [11]. In these cases, amivantamab induced severe erosive and necrotic lesions on the scalp and face that required drug cessation or dose modification while continuing treatment with doxycycline, prednisone, topical corticosteroids, or retinoids. George et al. reported extensive scalp involvement under the description “erosive pustular dermatosis-like eruption” [19]; however, this differs from the idiopathic neutrophilic erosive pustular dermatosis described by Pye et al. [20] and analyzed by Tomasini and Michelerio [21]. In the general population, erosive pustular dermatosis is rare, idiopathic, neutrophil-driven, and develops over months on atrophic or sun-damaged skin. In contrast, amivantamab-associated necrotic scalp lesions develop rapidly, likely due to EGFR-MET inhibition and recruitment of macrophages and neutrophils, suggesting a possible pathophysiological mechanism. Consequently, we propose that these lesions can be mitigated with prophylactic management prior to therapy initiation, including moisturizers, sunscreen, topical clindamycin, topical ivermectin, and oral tetracyclines (Table 1 and Table 2). One of our cases supports the effectiveness of this prophylactic approach in reducing the severity of dermatologic adverse events.

The COCOON phase 2 trial evaluated a dermatologic prophylactic regimen in the same patient population as the MARIPOSA trial. The regimen included oral doxycycline or minocycline for 12 weeks, 1% topical clindamycin daily on the scalp, 4% chlorhexidine daily on fingernails and toenails for 12 months, and daily use of ceramide-based moisturizers. This prophylactic approach significantly reduced the incidence of grade ≥ 2 dermatologic adverse events by 50% during the first 12 weeks, reduced grade ≥ 3 events by over 50%, and resulted in a three-fold decrease in moderate-to-severe scalp involvement [13].

Based on our experience with EGFR inhibitor-associated cutaneous eruptions, we believe that the timing, anatomic distribution, and severity of dermatologic adverse events are characteristic, reflecting the potent antitumor activity of amivantamab+lazertinib therapy. We believe that the severe papulopustular eruptions and necrotic folliculitis observed in these patients represent the more severe end of a recognized toxicity spectrum, rather than a distinct clinical entity. Prevention is critical not only to reduce the severity of these adverse events but also to minimize the risk of permanent complications, such as scarring, particularly in facial and scalp lesions.

Recent studies on EGFR inhibitor-associated alopecia have highlighted the involvement of the JAK-STAT1 signaling pathway. Dysfunctional EGFR signaling leads to fibrotic destruction of hair follicles mediated by interferon-gamma-expressing natural killer cells and cytotoxic T cells [22]. These findings suggest a potential role for Janus kinase (JAK) inhibitors in preventing or reversing scarring induced by anti-EGFR therapy in oncologic patients.

## 4. Conclusions

In conclusion, the novel combination of amivantamab and lazertinib, despite its promising efficacy in EGFR-mutated NSCLC, is frequently associated with dermatologic adverse events, particularly involving the face and scalp. These cutaneous reactions appear to develop more rapidly and severely than those typically observed with EGFR inhibitors. Effective management requires prompt recognition, accurate severity assessment, and tailored interventions, including corticosteroids, antibiotics, and other anti-inflammatory agents. In some cases, dose modification or drug cessation may be necessary.

Prophylactic measures are critical, as they can significantly reduce the severity of dermatologic side effects. Involvement of dermatologists in the care of patients receiving these targeted therapies is essential not only for diagnosing and managing complex skin reactions but also for implementing proactive strategies that support treatment adherence and improve oncologic outcomes.

## Figures and Tables

**Table 1:** Dermatologic interventional treatment suggestions based on the cited publications and our clinical experience (topical and systemic treatments can be combined regardless of the severity).

Topical	Systemic	Necrotic Lesions
Clindamycin emulsion or gel	Doxycycline 100 mg twice daily	Hydrogen peroxide soaks
Metronidazole cream	Tetracycline 500 mg twice daily	Iodine soaks
Ivermectin cream	Minocycline 50–100 mg twice daily	Mupirocin cream
Mupirocin cream or ointment	Isotretinoin 0.1–0.3 mg/kg per day	Chlorhexidine washes
Hydrocortisone cream or lotion	Acitretin 10 mg per day	Timolol solution Aluminum acetate soaks Silver nitrate soaks Silver sulfathiazole cream Betamethasone with gentamicin cream
Corticosteroids: fluocinolone, triamcinolone, prednisone, clobetasol	Antihistamines Prednisone 10 mg/day to 0.5 mg/kg/day (different doses)	
	Dapsone 25–50 mg per day Cefuroxime 500 mg twice daily	

**Table 2:** Preventive management suggestions for anti-EGFR therapy dermatologic adverse events [6,8,13].

Cleansers	Moisturizers and Photoprotection	Prophylaxis
Gentle cleanser close to skin pH (5.0) with lipid replenishing ingredients	Emollients “plus”	Doxycycline 100–200 mg per day
	Moisturizers containing ceramides, niacinamide, shea butter, and urea (low percent, 2–3%)	Tetracycline 250–500 mg per day
	Sunscreen with UV broad spectrum (UVA/UVB) filters to prevent possible post-inflammatory hyperpigmentation in darker phototypes	Topical clindamycin 1% lotion on the scalp daily
		Chlorhexidine 4% on the fingernails and toenails daily

Abbreviations: EGFR, epidermal growth factor receptor; UV, ultraviolet.

## Data Availability

All further inquiries should be directed to the corresponding author.

## Abbreviations

The following abbreviations are used in this manuscript:

EGFR	Epidermal growth factor receptor
NSCLC	Non-small cell lung cancer
MET	Mesenchymal–epithelial transition
TKI	Tyrosine kinase inhibitor
FDA	Food and Drug Administration
NCI CTCAE	National Cancer Institute Common Terminology Criteria for Adverse Events
UV	Ultraviolet
IL-1	Interleukin-1
TNF-α	Tumor necrosis factor-alpha
IRRs	Infusion-related reactions
AST	Aspartate aminotransferase
JAK-STAT1	Janus kinase signal transducer and activator of transcription 1
JAK	Janus kinase

## References

[1] Cho B.C., Kim D.W., Spira A.I., Gomez J.E., Haura E.B., Kim S.W., Sanborn R.E., Cho E.K., Lee K.H., Minchom A. (2023). Amivantamab plus lazertinib in osimertinib-relapsed EGFR-mutant advanced non-small cell lung cancer: A phase 1 trial. Nat. Med..

[2] FDA Approves Lazertinib with Amivantamab-Vmjw for Non-Small Lung Cancer. https://www.fda.gov/drugs/resources-information-approved-drugs/fda-approves-lazertinib-amivantamab-vmjw-non-small-lung-cancer.

[3] Cho B.C., Lu S., Felip E., Spira A.I., Girard N., Lee J.S., Lee S.H., Ostapenko Y., Danchaivijitr P., Liu B. (2024). Amivantamab plus Lazertinib in Previously Untreated EGFR-Mutated Advanced NSCLC. N. Engl. J. Med..

[4] Lacouture M.E. (2006). Mechanisms of cutaneous toxicities to EGFR inhibitors. Nat. Rev. Cancer.

[5] Braden R.L., Anadkat M.J. (2016). EGFR inhibitor-induced skin reactions: Differentiating acneiform rash from superimposed bacterial infections. Support. Care Cancer.

[6] Hofheinz R.D., Deplanque G., Komatsu Y., Kobayashi Y., Ocvirk J., Racca P., Guenther S., Zhang J., Lacouture M.E., Jatoi A. (2016). Recommendations for the Prophylactic Management of Skin Reactions Induced by Epidermal Growth Factor Receptor Inhibitors in Patients With Solid Tumors. Oncologist.

[7] Boone S.L., Rademaker A., Liu D., Pfeiffer C., Mauro D.J., Lacouture M.E. (2007). Impact and management of skin toxicity associated with anti-epidermal growth factor receptor therapy: Survey results. Oncology.

[8] Dreno B., Khosrotehrani K., De Barros Silva G., Wolf J.R., Kerob D., Trombetta M., Atenguena E., Dielenseger P., Pan M., Scotte F. (2023). The role of dermocosmetics in the management of cancer-related skin toxicities: International expert consensus. Support. Care Cancer.

[9] Vezzoli P., Marzano A.V., Onida F., Alessi E., Galassi B., Tomirotti M., Berti E. (2008). Cetuximab-induced acneiform eruption and the response to isotretinoin. Acta Derm. Venereol..

[10] Papoui E., Papastavrou E., Merkouris A., Charalambous A. (2021). The extent to which the last decade has yielded additional treatment options for EGFR-associated rash besides classic treatment with antibiotics and corticosteroids—A systematic review. Eur. J. Oncol. Nurs..

[11] Nowaczyk J., Fret K., Kaminska-Winciorek G., Rudnicka L., Czuwara J. (2023). EGFR inhibitor-induced folliculitis decalvans: A case series and management guidelines. Anticancer Drugs.

[12] Lacouture M., Sibaud V. (2018). Toxic Side Effects of Targeted Therapies and Immunotherapies Affecting the Skin, Oral Mucosa, Hair, and Nails. Am. J. Clin. Dermatol..

[13] Cho B.C., Girard N., Sauder M.B., Feldman J., Li W., Bozorgmehr F., Mak M., Smith J., Simoes J., Mahadevia P. (2024). P3.12D.04 Enhanced vs Standard Dermatologic Management with Amivantamab-Lazertinib in Advanced NSCLC: Phase 2 COCOON Study. J. Thorac. Oncol..

[14] Lacouture M.E., Basti S., Patel J., Benson A. (2006). The SERIES clinic: An interdisciplinary approach to the management of toxicities of EGFR inhibitors. J. Support. Oncol..

[15] Brodell L.A., Hepper D., Lind A., Gru A.A., Anadkat M.J. (2013). Histopathology of acneiform eruptions in patients treated with epidermal growth factor receptor inhibitors. J. Cutan. Pathol..

[16] Yang J.C., Lu S., Hayashi H., Felip E., Spira A.I., Girard N., Kim Y.J., Lee S.H., Ostapenko Y., Danchaivijitr P. (2025). Overall Survival with Amivantamab-Lazertinib in EGFR-Mutated Advanced NSCLC. N. Engl. J. Med..

[17] Ceylan F., Sonmez G., Tenekeci A.K., Unal A.A., Sendur M.A.N. (2025). Comprehensive management strategies for amivantamab-induced toxicities and review of the literature. J. Oncol. Pharm. Pract..

[18] Belzer A., Nguyen M.O., Talsania A., Haldas J., Smith J., Leventhal J.S. (2023). Spectrum of Dermatologic Adverse Events Associated With Amivantamab Use. JAMA Dermatol..

[19] George M.N., Seervai R.N.H., Chon S.Y. (2024). Erosive pustular dermatosis-like eruption of the scalp secondary to amivantamab: A case series. JAAD Case Rep..

[20] Pye R.J., Peachey R.D., Burton J.L. (1979). Erosive pustular dermatosis of the scalp. Br. J. Dermatol..

[21] Tomasini C., Michelerio A. (2019). Erosive pustular dermatosis of the scalp: A neutrophilic folliculitis within the spectrum of neutrophilic dermatoses: A clinicopathologic study of 30 cases. J. Am. Acad. Dermatol..

[22] Strobl K., Klufa J., Jin R., Artner-Gent L., Krauss D., Novoszel P., Strobl J., Stary G., Vujic I., Griss J. (2024). JAK-STAT1 as therapeutic target for EGFR deficiency-associated inflammation and scarring alopecia. EMBO Mol. Med..

